# Liquid biopsy in cancer drug resistance: real-time monitoring, mechanistic insights, and translational applications

**DOI:** 10.3389/fimmu.2026.1795789

**Published:** 2026-04-02

**Authors:** Yiyang Li, Zixuan Liu, Benliang Yuan, Tianshuai Zhang, Xiaoming Zhu, Leqi Zhou, Wei Zhang, Guanyu Yu

**Affiliations:** Department of Colorectal Surgery, Shanghai Changhai Hospital, Shanghai, China

**Keywords:** cancer drug resistance, circulating tumor cells, circulating tumor DNA, extracellular vesicles, liquid biopsy, precision medicine, real-time monitoring

## Abstract

Liquid biopsy provides a non-invasive approach for cancer detection. It examines tumor-associated components in blood and other body fluids, including circulating tumor cells (CTCs), circulating tumor DNA (ctDNA), and extracellular vesicles (EVs, including exosomes). This technology is transforming cancer diagnosis, prognosis, and therapy monitoring. This review summarizes recent advancements in liquid biopsy technologies, particularly focusing on circulating tumor components such as ctDNA, CTCs, and EVs. This review emphasizes the role of liquid biopsy in the early detection of drug resistance and therapeutic response monitoring. A primary emphasis is on how liquid biopsy detects various forms of drug resistance. It evaluates treatment efficacy, monitors alterations in tumor genomics over time, and assists in customizing therapy for people. We also discuss the integration of liquid biopsy with innovative technologies such as artificial intelligence and big data. The objective is to provide practical guidance for the future development of precision oncology.

## 1 Introduction

Liquid biopsy is revolutionizing oncological practice as a minimally invasive complement to conventional tissue biopsies. By interrogating circulating tumor-derived components—circulating tumor DNA (ctDNA), circulating tumor cells (CTCs), and extracellular vesicles (EVs, including exosomes)—it enables serial sampling to monitor tumor burden, clonal evolution, and emerging resistance over time. In clinical settings, this supports assessment of treatment response, detection of minimal residual disease (MRD), and identification of actionable alterations when tissue is unavailable or unsafe to obtain (1).

Conventional tissue biopsies are invasive and limited in sampling capacity, making them inadequate for accurately monitoring tumor dynamics over time. In contrast, liquid biopsy overcomes these obstacles (2). Liquid biopsy plays a pivotal role in precision medicine, as it can detect targetable mutations and identify treatment resistance at an early stage, thereby improving patient prognosis.

**Figure 1:**
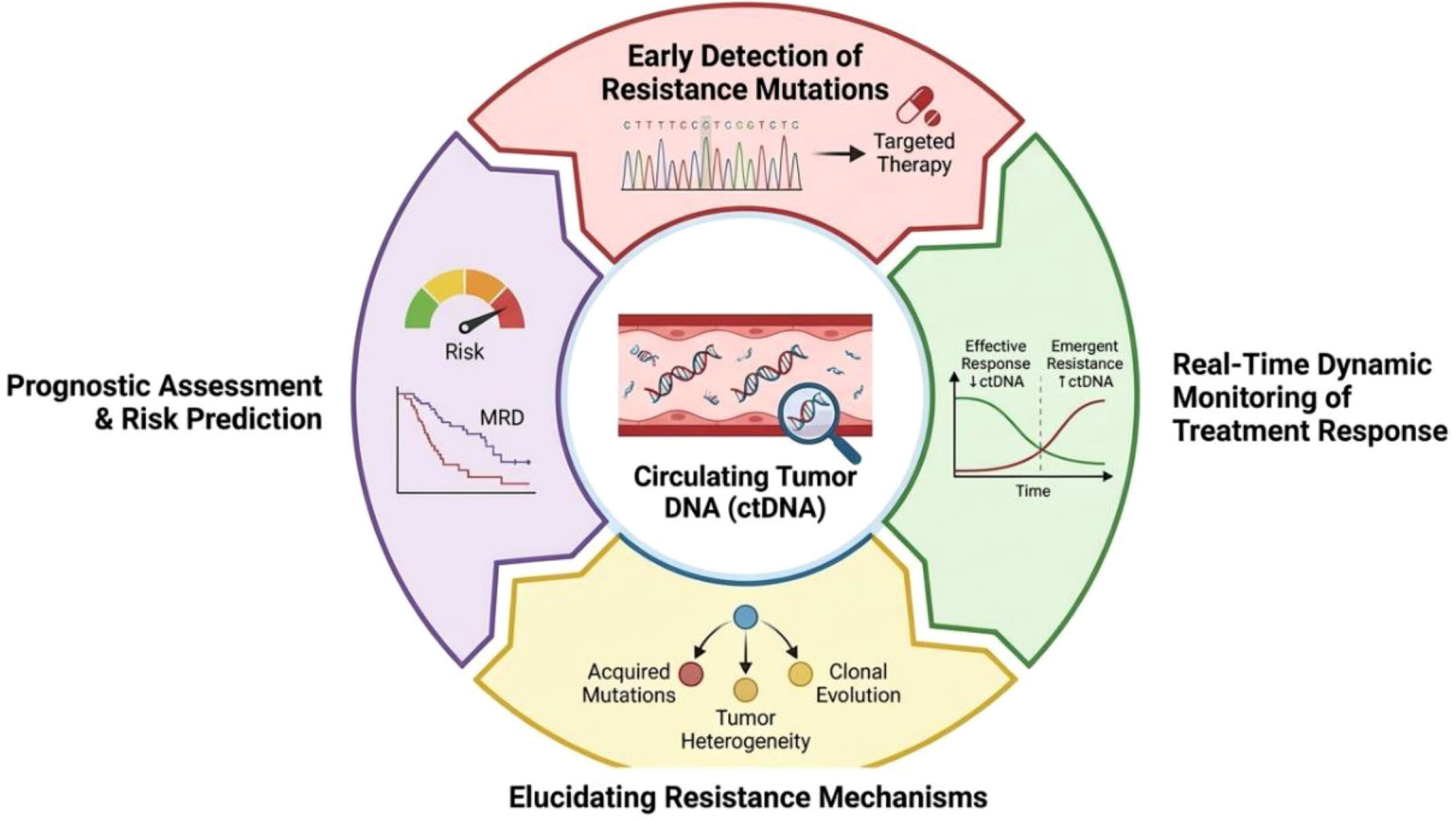
Overview of the clinical utilities of liquid biopsy for cancer drug resistance (ctDNA shown as a representative analyte). Serial sampling enables (i) early detection of resistance-associated mutations to guide therapy selection, (ii) real-time monitoring of treatment response and emergent resistance, (iii) prognostic assessment including minimal residual disease (MRD) and recurrence risk prediction, and (iv) elucidation of resistance mechanisms such as tumor heterogeneity, acquired mutations, and clonal evolution.

Liquid biopsy serves a crucial role in identifying cancer recurrence and assessing therapeutic efficacy. ctDNA dynamics often correlate with treatment response, and serial measurement can provide earlier indications of response or progression than imaging alone (3). For example, in EGFR-mutant NSCLC, plasma ctDNA testing for acquired T790M can facilitate timely switching to osimertinib, while a negative plasma result should be interpreted cautiously and may warrant tissue confirmation where feasible (4, 5). Clinical studies have validated the utility of liquid biopsy in identifying biomarkers associated with therapeutic resistance, recognition of which is essential for developing improved strategies for advanced cancer management (6). The major clinical utilities of liquid biopsy for cancer drug resistance are summarized in **Figure 1**.

**Figure 2:**
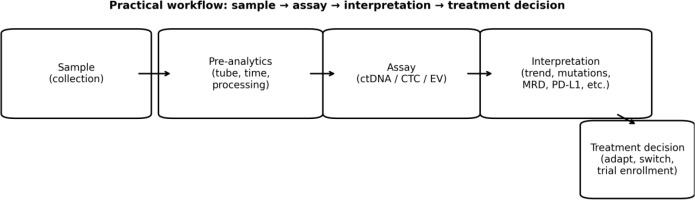
A clinically oriented workflow from sample collection to assay selection, integrated interpretation, and treatment decision-making for resistance monitoring using liquid biopsy analytes.

**Figure 3:**
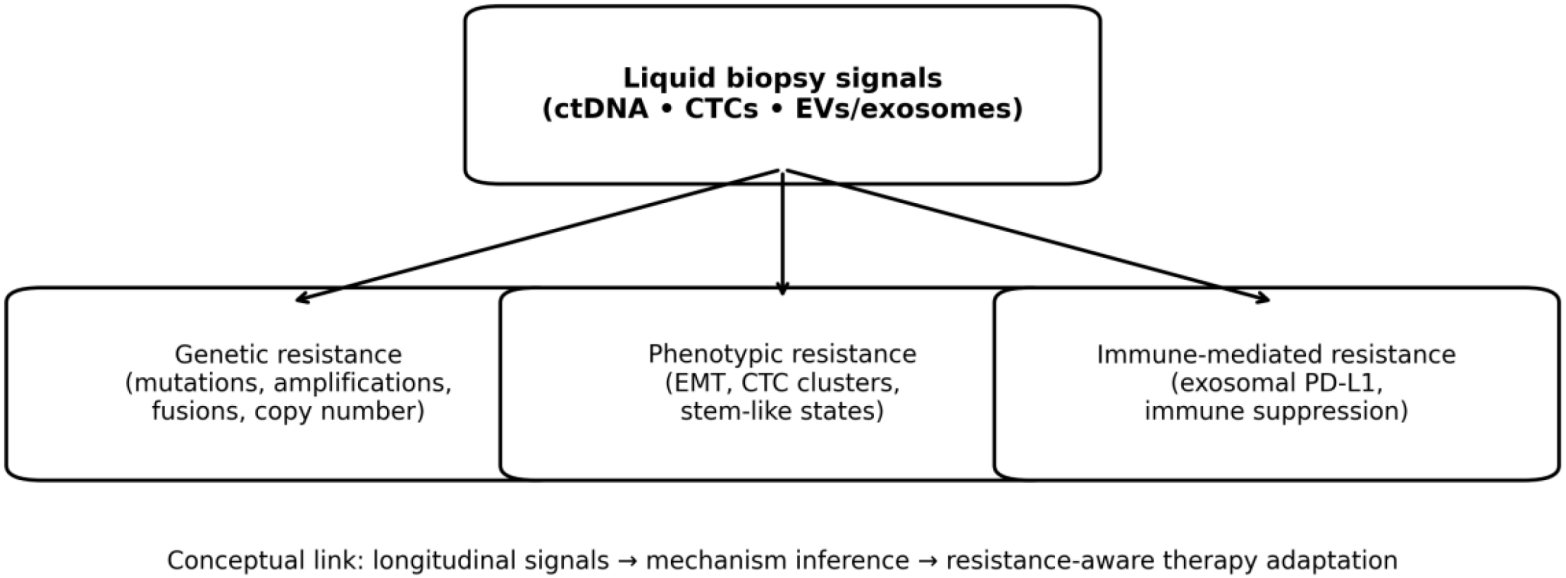
Conceptual diagram linking longitudinal liquid biopsy signals (ctDNA, CTCs, and EVs/exosomes) to major resistance mechanisms (genetic, phenotypic, and immune-mediated), supporting resistance-aware therapy adaptation.

Although liquid biopsy presents great potential for clinical application, considerable challenges remain. This includes maintaining uniform methodologies, analyzing intricate data, and translating findings into practical decision-making. Standardized clinical workflows and integrated interpretation across analytes are needed to ensure reproducibility and actionability (**Figure 2**), and mechanistic mapping of liquid biopsy signals to resistance biology can guide hypothesis-driven interventions (**Figure 3**) (7). As research uncovers further applications and advantages, liquid biopsy is poised to become a fundamental component of modern cancer treatment, as highlighted by several landmark reviews summarizing its evolution from research to clinical application (8–10).

## 2 Components and detection techniques of liquid biopsy

### 2.1 Circulating tumor cells

CTCs enter the bloodstream, serving as key indicators of cancer dissemination (metastasis) and tumor heterogeneity (11). CTCs, present in the bloodstream, can mimic the characteristics of the primary tumor. Furthermore, they exhibit malignant potential and distinct therapeutic responses, which facilitate metastatic spread (12, 13). CTCs promote metastasis by initiating the proliferation of secondary tumors in distant organs (11).

Analyzing CTCs elucidates the temporal evolution of malignancies, enabling physicians to monitor therapy efficacy and illness progression in real time (12, 13). Researchers have developed multiple methods to identify and examine CTCs. Principal techniques encompass immunoaffinity capture, microfluidic devices, and biophysical separation (14).

Immunofluorescence approaches employ particular antibodies to capture CTCs. Antibodies frequently target epithelial cell adhesion molecule (EpCAM), a membrane-associated protein. However, this approach possesses a significant limitation. Some CTCs undergo epithelial–mesenchymal transition (EMT), which increases their aggressiveness and drug resistance while reducing or eliminating EpCAM expression on their surface. Consequently, CTCs with little or absent EpCAM evade detection by EpCAM-targeting techniques (14). In contrast, microfluidic devices have the ability to capture a greater diversity of cellular phenotypes by separating CTCs based on their size and deformability (15).

CTC genomic, transcriptomic, and proteomic studies illuminate tumor biology, therapeutic responses, and drug resistance (11, 12). For instance, CTC expression profiles and mutations can inform treatment and patient outcomes. Dynamic CTC monitoring detects MRD following treatment and evaluates therapy efficacy (13).

Although CTC research has made progress, challenges remain in standardizing isolation and analytical methodologies, as well as in better integrating CTC data into clinical practice (12, 13). As liquid biopsy technology improves, CTC profiling could reveal critical insights into tumor heterogeneity and drug resistance, enabling customized cancer treatment and improved patient outcomes (11–13).

### 2.2 Circulating tumor DNA

The analysis of ctDNA enables clinicians to monitor tumor progression and treatment effectiveness without the need for surgical intervention. As a carrier of tumor-specific mutations, ctDNA exhibits both sensitivity and specificity in cancer diagnosis. This characteristic aids in identifying genetic alterations that invasive and restricted tissue biopsies overlook. ctDNA, which circulates in the bloodstream, reflects tumor genetic alterations as cancers develop resistance or respond to therapy (11, 16).

The detection of ctDNA has progressed through digital PCR, NGS, and multiplex targeted sequencing. Digital PCR is highly sensitive for detecting low-frequency ctDNA mutations, enabling early identification and monitoring of MRD (17, 18). Next-generation sequencing (NGS) can analyze a greater number of genomes and identify numerous mutations, offering a comprehensive overview of the tumor’s genetic alterations. This technology is enhanced by multiplex targeted sequencing, which concurrently evaluates numerous genes. This facilitates the identification of mutations for targeted therapy (19, 20).

Monitoring fluctuations in ctDNA levels throughout therapy can significantly enhance our comprehension of therapeutic efficacy and the emergence of drug resistance. Research indicates that monitoring ctDNA can early identify treatment efficacy, enabling physicians to promptly modify regimens. Effective cancer treatment typically coincides with a substantial reduction in ctDNA levels. Elevated ctDNA levels may signify cancer progression or treatment resistance (17, 21). ctDNA could act as the biomarker for individualized cancer treatment, which enables physicians to monitor tumor treatment responses in real time, and allows them to customize treatments according to each patient’s distinct tumor characteristics. Cancers may acquire genetic alterations that confer treatment resistance over time; however, ctDNA testing can detect these mutations early. Rapid identification of mutations enables physicians to modify treatment strategies to combat with cancer treatment resistance, hence enhancing patient outcomes (11, 17). This proactive strategy is particularly important for diseases such as colorectal and lung cancer, which are recognized for their capacity to evolve and adapt over time (18, 20).

ctDNA provides real-time information about tumor genetics, and new detection methods make it a key part of the customized cancer treatment strategy. As ongoing research improves detection technologies and expands their clinical use, ctDNA will play an increasingly important role in cancer management, enabling more precise and effective treatment planning.

### 2.3 Extracellular vesicles and exosomes

Tumor cells communicate via extracellular vesicles (EVs), a heterogeneous population of membrane-bound particles released into biofluids. Exosomes are a subtype of small EVs and can transport diverse cargos, including microRNAs (miRNAs) that regulate proliferation and apoptosis, as well as long non-coding RNAs (lncRNAs) and circular RNAs (circRNAs) that modulate gene expression and stress responses (22, 23).

These observations support EVs/exosomes as both biomarkers and molecular communication vehicles, providing a window into tumor dynamics and therapy responses.

EV isolation and purification are essential for clinical use in liquid biopsy. In practice , many workflows enrich mixed EV populations rather than pure exosomes, which can complicate cross-study comparisons. Common isolation technologies include differential ultracentrifugation, immunocapture, and microfluidics. Ultracentrifugation is widely used but can be time-consuming and may co-isolate proteins or other vesicle subtypes. Immunocapture uses antibodies against EV surface markers for more selective enrichment, although marker choice and reagent availability can limit robustness. Clinical applications favor rapid and efficient isolation with minimal sample processing, and microfluidic techniques are increasingly promising (24, 25).

EV-associated nucleic acids, proteins, and lipids show promise for understanding malignancy and drug resistance, as these molecules participate in cellular communication and tumor progression. Tumor-derived EVs/exosomes can reflect the tumor’s genetic and epigenetic landscape, supporting non-invasive monitoring of tumor dynamics and therapy effectiveness. EV-associated miRNAs and other nucleic acids can propagate drug-resistance traits between cells, adding complexity to cancer treatment (26, 27). These features also highlight EV roles in the tumor microenvironment (TME) and in resistance-related intercellular signaling .

EV proteins and lipids can themselves drive malignant behavior and therapy failure. Tumor-derived EVs/exosomes (often termed TEXs) have been shown to carry oncogenic proteins such as EGFRvIII and transfer functional receptor signaling to recipient cells, promoting proliferation, invasion, and angiogenesis (28). Moreover, PD-L1 presented on the surface of TEXs can suppress T cell function systemically and has been implicated in resistance to PD-1/PD-L1 immune checkpoint blockade in several cancer types (29). Finally, EV lipids can reprogram recipient-cell metabolism and membrane signaling (e.g., by modulating lipid rafts and downstream survival pathways), thereby supporting metastatic dissemination and contributing to chemoresistance (30).

In conclusion, EVs/exosomes transport biologically active nucleic acids, proteins, and lipids, making them a promising component of liquid biopsy for resistance research and, potentially, clinical monitoring. However, improved isolation methods and standardized reporting are necessary for reliable translation. To facilitate comparison and understanding of key liquid biopsy components, **Table 1** summarizes major detection methods, advantages, limitations, and clinical implications across analytes (10).

**Table 1:** Comparison of liquid biopsy components: CTCs, ctDNA, and extracellular vesicles (EVs)/exosomes.

Biomarker	Origin	Key detection techniques	Advantages	Limitations	Clinical significance (e.g., in drug resistance)
Circulating Tumor Cells (CTCs)	Detach from primary or metastatic tumors and enter the bloodstream.	Immunoaffinity capture (e.g., anti-EpCAM) Microfluidic devices (size/deformability-based) Biophysical separation methods	Provide whole living cells for functional analysis (genomic, transcriptomic, proteomic). Directly indicate metastatic potential and tumor heterogeneity. Can be cultured *ex vivo* for drug testing.	Extremely rare in blood, requiring highly sensitive techniques. EpCAM-based methods miss CTCs undergoing Epithelial-Mesenchymal Transition (EMT). Lack of standardized isolation and analysis protocols.	Heterogeneity analysis: Single-cell analysis can identify pre-existing resistant subclones.
					EMT Monitoring: CTCs with mesenchymal markers are associated with increased aggressiveness and therapy resistance. Cluster
					Detection: CTC clusters have higher metastatic potential and may be more resistant to therapy.
Circulating Tumor DNA (ctDNA)	Released into bloodstream from apoptotic or necrotic tumor cells.	Digital PCR (dPCR) Next-Generation Sequencing (NGS) Multiplex Targeted Sequencing	High specificity for tumor-specific mutations. Allows for real-time monitoring of tumor genomics and dynamics. Less invasive than tissue biopsy, enabling frequent sampling.	Can be fragmented and present in low abundance, especially in early-stage cancer. May not fully capture tumor heterogeneity compared to whole cells. Challenges in distinguishing clonal hematopoiesis mutations.	Early Resistance Detection: Identifies emerging resistance mutations (e.g., EGFR T790M in NSCLC) during targeted therapy.
					MRD Monitoring: Detects minimal residual disease, predicting relapse.
					Therapy Response: Decreasing ctDNA levels indicate treatment efficacy; rising levels suggest resistance or progression.
Extracellular Vesicles (EVs; including exosomes)	Secreted by various cell types, including tumor cells, as nanosized extracellular vesicles (a subset termed exosomes).	Differential ultracentrifugation/size-exclusion chromatography Immunocapture (antibodies against EV surface markers) Microfluidic isolation and on-chip analysis	Contain a rich cargo of nucleic acids (miRNA, lncRNA, circRNA), proteins, and lipids reflective of the parent cell. Highly stable in circulation and abundant in bodily fluids. Play an active role in cell-cell communication and TME modulation.	Isolation of pure EV subpopulations is technically challenging and time-consuming. Standardization of isolation, storage, and normalization strategies remains limited. Complex cargo analysis often requires multi-omics and robust bioinformatics.	Resistance mechanisms: EV cargo can transfer drug-resistant traits (e.g., miRNAs, proteins) to sensitive cells, conferring resistance.
					TME and immune modulation: tumor-derived EVs can remodel the microenvironment and promote immune escape.
					Biomarker source: EV nucleic acids and proteins can support monitoring of therapyresponse and emerging resistance when integrated with ctDNA/CTC signals.

While ctDNA, CTCs, and EVs/exosomes are often discussed separately, they provide complementary layers of resistance biology in clinical oncology. ctDNA is most informative for tracking genomic mechanisms (e.g., emergent resistance mutations and clonal dynamics), whereas CTCs offer single-cell and phenotypic resolution (EMT states, clusters, and functional assays). EVs/exosomes can reflect tumor-microenvironment crosstalk and immune-mediated mechanisms (e.g., immunomodulatory cargo) that may not be captured by ctDNA alone. Discordant results can occur—for example, ctDNA may be negative in low-shedding settings while CTCs/EVs remain detectable, or ctDNA may reveal a resistance mutation before changes in CTC counts are apparent. A pragmatic strategy is to interpret biomarker discordance in clinical context, verify pre-analytical quality, repeat sampling when needed, and pursue tissue confirmation when results would change therapy (**Figure 2**).

## 3 Clinical applications of liquid biopsy in efficacy monitoring

### 3.1 Real-time dynamic monitoring of treatment response and emergent resistance

The measurement of CTCs and ctDNA through liquid biopsy has enabled serial, minimally invasive monitoring of treatment responses across chemotherapy, targeted therapy, and immunotherapy settings (31, 32). Longitudinal changes in ctDNA variant allele fractions or tumor fraction can precede radiographic changes, providing earlier indications of response or molecular progression, whereas CTC analyses can add phenotypic context (e.g., EMT programs and cluster formation) that may help explain heterogeneous sensitivity to therapy (33).

A clinically established example is EGFR-mutant non-small cell lung cancer (NSCLC): emergent EGFR T790M detected in plasma ctDNA can prompt a switch to osimertinib, and negative plasma results should be interpreted cautiously and followed by tissue testing when feasible because plasma sensitivity is imperfect (4, 5, 34). Similar serial ctDNA frameworks are being explored for additional actionable alterations (e.g., ESR1 in breast cancer and RAS in colorectal cancer) to guide timely therapy adaptation and to reduce exposure to ineffective treatment (35).

Liquid biopsy can also support immunotherapy monitoring. Early decreases in ctDNA during immune checkpoint blockade have been associated with clinical benefit in several tumor types, while rising ctDNA may indicate early resistance or hyperprogression in select contexts (36, 37). EV/exosomal markers such as PD-L1 are mechanistically linked to immune suppression; however, reported clinical correlations have been heterogeneous across studies, partly reflecting variability in EV isolation, PD-L1 quantification, and normalization strategies, underscoring the need for standardized assays before routine decision-making (9, 38).

Importantly, monitoring alone does not guarantee improved outcomes unless linked to validated intervention strategies. In SWOG S0500, early chemotherapy switching based solely on persistently elevated CTCs after one cycle did not improve overall survival, highlighting the need for biomarker-guided decisions to be tested prospectively (39). Ongoing efforts therefore focus on integrating liquid biopsy signals with clinical and imaging data, defining actionable thresholds, and standardizing timing for therapy modification within prospective trials (40).

In addition to ctDNA and CTCs, extracellular vesicles (EVs; including exosomes) are emerging as valuable biomarkers and mechanistic mediators of treatment response and drug resistance. Tumor-derived EVs/exosomes (TEXs) carry diverse molecular cargos—including miRNAs, lncRNAs, proteins, and lipids—that reflect the physiological state of their parent cells. EV-associated miRNAs such as miR-21 and miR-1246 have been reported to promote chemoresistance in breast and lung cancers by regulating apoptotic and PI3K/AKT signaling pathways (41). In glioblastoma, EVs bearing the mutant receptor EGFRvIII can transmit oncogenic signaling to neighboring cells, fostering tumor progression and treatment resistance (28). These findings underscore that integrating EV analysis into clinical liquid biopsy may provide a more comprehensive assessment of tumor evolution, immune escape, and therapeutic efficacy when interpreted alongside ctDNA and CTC readouts.

### 3.2 Prognostic assessment and recurrence risk prediction

The use of liquid biopsy technology, particularly the detection of CTCs and ctDNA, has greatly improved the ability to predict the outcome of cancer treatment. The mutational burden of ctDNA serves as a predictive indicator of recurrence risk. Elevated diagnostic CTC levels are associated with poorer prognosis. Research on breast cancer and colorectal cancer illustrates this correlation (42). Increased levels of CTCs are associated with reduced overall survival rates. The rates of metastasis are observed to rise in correlation with elevated counts of CTCs. ctDNA analysis serves as a valuable tool for monitoring the evolution of tumors. The assessment of treatment response offers significant advantages. Post-treatment detectable ctDNA serves as a predictor for recurrence. Persistent ctDNA after treatment indicates a higher risk of relapse, whereas undetectable ctDNA is associated with a more favorable prognosis (43, 44). The results indicate a strong case for clinical integration. CTC and ctDNA metrics facilitate the development of tailored strategies. Adaptive therapy focuses on the progression of disease over time.

Multiparametric analysis combining multiple biomarkers enhances the precision of prognostic assessments. The integration of biomarker evaluations yields enhanced insights. CTC enumeration serves as a valuable addition to ctDNA profiling. The evaluation of tumor biology is conducted with greater comprehensiveness. The reliability of treatment outcome forecasting is improving. Research on prostate cancer supports this methodology. Multimarker panels demonstrate superior performance compared to single biomarkers (45).

Incorporating these indicators into clinical workflows facilitates the early detection of tumor recurrences, and allows us to make proper adjustments to cancer treatment plans.

Alongside CTCs and ctDNA, other components of liquid biopsies, including extracellular vesicles (EVs; encompassing exosomes) and tumor-educated platelets, are increasingly recognized for their prognostic potential. Tumor-derived EVs transport molecular signatures indicative of their originating tumors and can be isolated from multiple body fluids. EV-based profiles have shown promise in forecasting treatment responses and recurrence risks in several malignancies, including ovarian and pancreatic cancers (46, 47). In addition, machine learning approaches applied to multi-omic liquid biopsy data may improve risk stratification and recurrence prediction, facilitating more personalized patient management strategies.

Advancements in liquid biopsy technologies, particularly the analysis of CTCs and ctDNA, facilitate real-time observation of tumor dynamics and incorporate multi-parametric analyses, which may improve the precision of prognostic evaluations and predictions of recurrence risk.

### 3.3 Application examples in different tumor types

Liquid biopsy evaluates treatment efficacy for solid tumors, such as prostate, breast, non-small cell lung, and colorectal malignancies. In prostate cancer, surveillance leveraging both CTCs and ctDNA provides dynamic insights into tumor behavior. For instance, fluctuations in ctDNA levels can signal disease progression or treatment response, thereby informing timely therapeutic adjustments (48). In breast cancer, particularly in metastatic settings, liquid biopsy is widely used. The detection of CTCs and ctDNA serves as an indicator of tumor burden and significantly improves the assessment of therapeutic response, facilitating more informed clinical decisions (49). Liquid biopsy plays a primary role in managing NSCLC, where traditional biopsies are often limited by anatomical constraints and tumor location. This approach enables effective identification of driver gene mutations and advanced monitoring of disease progression (50). Colorectal cancer uses ctDNA analysis efficiently. Recurrence prediction is clinically useful (51– 52). Treatment response monitoring indicates efficacy. These applications demonstrate methodological versatility. Clinical importance extends across tumor types. Personalized treatment strategies show significant improvement.

Given the tumor-type specificity of biomarkers, different analytical approaches are often required due to underlying biological variances. As noted, prostate cancer management relies on ctDNA and CTCs to evaluate therapy efficacy by demonstrating tumor dynamics. The efficacy of therapy can be evaluated. Breast cancer uses CTCs and miRNAs, in which treatment response evaluation progresses and disease progression has been monitored (53). NSCLC diagnosis increasingly incorporates ctDNA analysis, particularly for detecting EGFR mutations, which directly guides targeted therapy customization (52). Colorectal cancer uses ctDNA and TEP analysis, which shows treatment outcome prediction has improved and MRD surveillance continues (11).

Thus, liquid biopsy is transforming cancer treatment by enabling non-invasive therapy monitoring and operationalizing early resistance detection across a wide spectrum of malignancies, accelerating the evolution of precision oncology.

## 4 Role of liquid biopsy in early drug resistance detection mechanisms

### 4.1 Early capture of drug resistance-related gene mutations

The identification of resistance mutations is essential for the success of targeted therapies, particularly in non–small cell lung cancer (NSCLC). ctDNA testing enables highly sensitive detection of driver gene mutations, such as EGFR, ALK, and KRAS, which are closely associated with targeted therapy resistance. Studies have confirmed ctDNA’s capability to detect resistance mutations that emerge during treatment. For example, the EGFR T790M mutation indicates resistance to tyrosine kinase inhibitors (TKIs). ctDNA analysis allows for its early detection, enabling timely therapeutic switching to third-generation TKIs such as osimertinib before clinical progression occurs (54). Dynamic mutation monitoring through ctDNA analysis informs treatment adjustments and reflects tumor genomic evolution, ultimately improving patient outcomes. Early detection of resistance mutations facilitates the selection of effective second-line therapies, avoids ineffective treatments, and enhances disease management. This approach underscores the importance of ctDNA as a non-invasive tool for real-time resistance testing and personalized oncology (55).

### 4.2 EV/exosome-mediated drug resistance mechanisms

Extracellular vesicles (EVs), particularly small EVs/exosomes, are nanosized vesicles secreted by various cell types and play a crucial role in intercellular communication. They contribute to drug resistance by transmitting miRNAs and lncRNAs that regulate signaling pathways related to therapy resistance. For instance, EV-associated miRNAs can modulate the PTEN/AKT signaling pathway, promoting cell survival and proliferation, thereby contributing to resistance against chemotherapeutic agents. EV cargo can also induce epithelial-mesenchymal transition (EMT), which enhances tumor invasiveness and is frequently associated with reduced therapeutic responsiveness; the transfer of specific miRNAs through EVs alters the expression of EMT-associated genes, resulting in a more aggressive and treatment-resistant phenotype (55, 56).

Tumor-derived EVs/exosomes (TEXs) play a pivotal role in mediating communication between cancer cells and the tumor microenvironment (TME). They influence the activity of surrounding stromal and immune cells, leading to phenotypic transformation and the establishment of supportive niches that promote tumor survival and drug resistance. TEXs remodel the stroma, accelerate tumor growth, and suppress antitumor immune responses, thereby enhancing the survival of resistant cancer cells. Consequently, conventional therapies encounter significant barriers imposed by the altered TME (57, 58).

EVs/exosomes further mediate drug resistance by transferring resistant phenotypes between cells. Drug-sensitive cells can acquire resistance after internalizing EVs derived from resistant cancer cells. This horizontal transfer of bioactive molecules—including proteins and nucleic acids—can reprogram recipient cells toward a resistant phenotype. Therefore, EVs represent both potential biomarkers of drug resistance and promising therapeutic targets, as modulating their biogenesis, uptake, or cargo may enhance treatment efficacy (59, 60).

In summary, tumor-derived EVs/exosomes can transport non-coding RNAs and proteins that regulate critical signaling pathways, modulate the TME, and mediate intercellular transfer of drug-resistant traits, collectively contributing to cancer treatment resistance. EV-mediated immune suppression (including via EV-associated PD-L1 in selected contexts) represents an additional resistance layer. While mechanistic evidence is strong, translation of specific EV biomarkers into clinical decision tools remains limited by assay heterogeneity and incomplete prospective validation, emphasizing the need for standardized isolation and quantification protocols (9, 57, 61).

### 4.3 CTC heterogeneity and drug resistance association

Treatment-induced stress and tumor cell adaptation contribute to the heterogeneity of CTCs. Genetic and phenotypic diversity among CTC subpopulations significantly affects their response to targeted therapies. Studies have shown that CTCs exhibit varying epithelial and mesenchymal markers during EMT. As tumor cells adapt to microenvironmental pressures and therapeutic exposure, they display differential sensitivity to treatment. In breast cancer, most CTCs display mesenchymal characteristics, which are associated with enhanced metastatic potential and increased treatment resistance (14).

Single-cell sequencing has revealed the presence of drug-resistant CTCs carrying distinct genetic and epigenetic alterations. In colorectal cancer, CTCs exhibit considerable genetic heterogeneity, including previously identified resistance-associated mutations (62). These findings highlight the crucial role of CTC heterogeneity in mediating drug resistance. Advanced transcriptomic profiling has identified resistance-related pathways that promote CTC survival and proliferation (63). Furthermore, CTC clusters demonstrate higher survival and metastatic capacity, complicating therapeutic response due to their collective protection from cytotoxic agents (15). Given their dynamic nature influenced by the TME and ongoing treatment, further research is needed to elucidate the precise role of CTCs in cancer progression and resistance. Enhanced molecular characterization and single-cell analysis may identify new therapeutic targets and support the development of more personalized treatment strategies.

## 5 Technical challenges and solutions strategies

### 5.1 Limitations of detection sensitivity and specificity

In liquid biopsies, detecting low-abundance CTCs and ctDNA has considerable challenges. Traditional approaches may not provide the necessary sensitivity to identify these cells or molecules. ctDNA released into the bloodstream during tumor cell apoptosis could become fragmented and degraded, which would lead to a restricted amount of intact DNA available for analysis. These may lead to missed opportunities for early diagnosis and adjustments in treatment strategies. Research demonstrates that the sensitivity of liquid biopsy tests varies considerably depending on the methodologies utilized, and different types of cancer exhibit ctDNA detection sensitivities between 50% and 75% (64, 65). Innovative solutions to current technical challenges in liquid biopsy are summarized in **Table 2**.

**Table 2:** Innovative solutions to liquid biopsy technical challenges.

Challenge	Emerging solution	Clinical impact	References
Low ctDNA abundance	Ultra-sensitive mutation detection (e.g., CRISPR-based assays and error-corrected sequencing)	Improved detection of low-frequency resistance mutations and MRD signals	(72, 73)
CTC heterogeneity	Microfluidic single-cell sorting	Analysis of CTC heterogeneity	(14, 15)
EV/exosome isolation	Standardized EV isolation (e.g., size-exclusion and microfluidic enrichment) with AI-enabled QC	Improved isolation efficiency, purity, and downstream biomarker reproducibility	(24, 61)
Pre-analytical variability	Standard operating procedures (SOPs), stabilizing tubes, controlled processing time, and external quality assessment	Reduced inter-laboratory variability and improved comparability across studies	(74–77)
Discordant biomarker signals	Integrated multi-analyte interpretation, repeat sampling, orthogonal assays, and tissue confirmation when actionable	More reliable treatment decisions and fewer false-negative/false-positive interpretations	(78)

Scientists are exploring innovative methods to improve the efficacy of liquid biopsy testing. High-throughput sequencing technology has improved the sensitivity of liquid biopsies. It detects actionable mutations that can aid clinicians in treatment decision-making (66, 67). Digital PCR can provide improved accuracy in measuring ctDNA and identify low-frequency mutations with high specificity (68, 69).

The utilization of AI algorithms significantly improves data analysis by detecting trends and increasing the precision of biomarker discovery. This will result in improved patient outcomes via prompt and personalized treatment strategies.

Despite these advancements, there are ongoing challenges in achieving standardization and maintaining the affordability of these technologies. High-throughput sequencing and digital PCR present significant challenges due to their considerable costs. The absence of standardized protocols for sample collection, processing, and analysis, could lead to variability in outcomes, which makes the interpretation of liquid biopsy results more complicated (70, 71). Recent studies indicate notable advancements in the development of detection methods that are both more sensitive and specific. Additionally, there is an emphasis on standardizing procedures and reducing costs, which enhances the future applicability of liquid biopsies in the management of cancer. .

### 5.2 Standardization and clinical validation

The implementation of digital PCR and NGS has the potential to significantly enhance the specificity and sensitivity of liquid biopsy testing, thereby influencing clinical decision-making (79). The variability in test results may be heightened due to unstandardized practices in sample collection, storage, and processing, along with other pre-analytical procedures (74). Disparities in results hamper liquid biopsy data interpretation and replication across clinical situations.

Multi- center clinical validation frameworks and harmonized standards are urgently needed to address these issues. The International Liquid Biopsy Standardization Alliance (ILSA) has promoted liquid biopsy in oncology globally by advocating for standardization (75). There are no comprehensive liquid biopsy standards that cover sample collection or interpretation. Such standards are needed to ensure the reliability of liquid biopsy assays in clinical practice , enable regulatory approval, and allow accurate research comparisons (76). Synchronizing laboratory techniques reduces inter-laboratory variability, which may increase liquid biopsy testing’s therapeutic relevance. Quality control and regular external quality reviews could improve the reliability of liquid biopsy findings. This would boost clinicians’ confidence in using these diagnostics for patient treatment (77).

In summary, the implementation of clinical validation and standardization is essential for the effective use of liquid biopsy.

### 5.3 Data interpretation and clinical decision support

Due to tumor variability and complex biomarker profiles, data extracted from liquid biopsies pose challenges for analysis and clinical interpretation. Tumor heterogeneity—variation across lesions, time, and cell states—can lead to discordant results between analytes (e.g., ctDNA versus CTCs/EVs) and between liquid biopsy and tissue biopsy, underscoring the need for context-aware interpretation and validation (78). Discordance may reflect biological factors (low tumor shedding, compartmentalized disease such as central nervous system involvement, or therapy-induced phenotypic shifts) as well as technical factors (pre-analytical handling, assay limits of detection, and contamination with non-tumor DNA including clonal hematopoiesis). A pragmatic clinical approach is to: (i) confirm pre-analytical quality and exclude common confounders; (ii) interpret trends rather than single time points; (iii) resolve discordant or unexpected results with repeat sampling and orthogonal assays; and (iv) pursue tissue confirmation when the result would alter therapy selection or trial eligibility.

Liquid biopsy data analysis benefits from the application of data analytics and machine learning. New technologies enable accurate predictive models in cancer biology. Machine learning algorithms help clinicians identify patterns and correlations in large datasets to improve treatment recommendations. A recent study used dynamic variables and longitudinal liquid biopsy data to predict stomach cancer therapy responses (80).

This approach accurately predicted therapeutic responses using tumor marker indices and cellular pictures, and shows how machine learning can improve liquid biopsy analysis and enable cancer-specific therapy plans.

Tumor biology and resistance mechanisms are better understood with genomic, transcriptomic, and proteomic data. Advanced bioinformatics methods help extract and analyze liquid biopsy data to identify treatment outcome biomarkers. In early cancer detection, an automated multimodal framework surpassed conventional methods (81). This method improves the predictive accuracy of clinical models. To implement liquid biopsy technology in clinical practice , oncologists, data scientists, and bioinformaticians should collaborate.

In summary, the interpretation of data from liquid biopsies presents challenges related to tumor heterogeneity and biomarker complexity. However, the incorporation of big data analytics and machine learning provides a means to improve clinical decision support. Utilizing these technologies enables healthcare providers to enhance the precision and dependability of liquid biopsy analyses, thereby facilitating more effective and individualized treatment approaches for cancer patients.

## 6 Future development directions and clinical translation prospects

### 6.1 Multi-omics combined liquid biopsy

Multi-omics integration is increasingly used to address two central challenges highlighted in this review: tumor heterogeneity and the complexity of resistance mechanisms. In practice , joint profiling of ctDNA (genomic alterations), CTCs (cellular phenotypes), and EVs/exosomes (intercellular signaling cargo) can provide a more comprehensive view of tumor evolution than any single analyte alone. By leveraging harmonized multi-omic pipelines, longitudinal sampling may reduce uncertainty from discordant single-marker results and improve detection of emerging resistance or minimal residual disease. Computational frameworks, including machine learning applied to integrated multi-omic features, can identify composite signatures associated with response and resistance, thereby enabling more clinically actionable monitoring strategies (82–85).

### 6.2 Deep integration of artificial intelligence and liquid biopsy

Artificial intelligence (AI) can help translate high-dimensional, multi-analyte liquid biopsy data into clinically actionable information by addressing data complexity, noise, and longitudinal variation. Machine learning and deep learning methods can integrate ctDNA, CTC, and EV-derived features with clinical and imaging variables to improve sensitivity and robustness, especially in settings where single-analyte signals are sparse. Beyond pattern recognition, AI-enabled models can support resistance monitoring by learning trajectories that precede radiographic progression and by prioritizing which alterations are likely to be actionable or biologically meaningful. However, prospective validation and transparent reporting of model performance are essential before AI-guided decision support can be broadly implemented (86–88).

Intelligent platforms facilitate the integration of liquid biopsy technologies into routine clinical practice , such as sample collection and data analysis, thereby improving the accessibility and practicality of liquid biopsy for healthcare providers. AI algorithms can automate the classification of liquid biopsy samples, thereby decreasing the time and labor required for manual analysis. This automation is advantageous in contexts requiring swift decision-making, such as cancer care, where prompt treatments can greatly influence patient outcomes (10).

AI can standardize liquid biopsy methods, ensuring consistent results across clinical settings. Standardization addresses variability and reliability concerns in diagnostic results, making liquid biopsy methods more widely accepted (89).

The potential of AI-driven liquid biopsy is not limited to diagnostics; it is also essential for the early detection of resistance and the monitoring of treatment efficacy. AI can identify changes in tumor dynamics and molecular profiles by analyzing longitudinal data from liquid biopsies, enabling clinicians to adjust treatment strategies in real-time. For example, the identification of particular mutations in ctDNA can suggest the emergence of resistance to targeted therapies, necessitating the implementation of treatment regimen modifications in a timely manner (10). Integration of AI with liquid biopsy technologies has a transformative impact on cancer management, promoting a more responsive and personalized healthcare paradigm.

In summary, the profound integration of AI with liquid biopsy is a substantial development in the field of cancer diagnostics and management. AI is on the brink of revolutionizing the clinical utility of liquid biopsy by improving detection sensitivity, streamlining clinical workflows and facilitating real-time monitoring of treatment responses. As research continues to develop, the synergistic potential of these technologies is expected to result in a paradigm shift in the diagnosis and treatment of cancer, as well as enhanced patient outcomes (86, 89).

### 6.2 Emerging biomarkers and technological innovations

The investigation of new biomarkers in liquid biopsy has garnered considerable attention, particularly for resistance monitoring where genomic alterations alone may be insufficient. Emerging biomarkers include EV/exosomal nucleic acids and proteins as well as circulating metabolites that reflect tumor metabolism and therapy-induced reprogramming. Recent studies have highlighted EV-associated RNAs and proteins as candidate markers of resistance in cancers such as breast and lung cancer (90, 91). In parallel, metabolomic profiles from blood or other biofluids may capture pathway-level adaptations linked to chemotherapy response or resistance; nevertheless, metabolomic findings can vary across platforms and cohorts and require rigorous standardization and clinical validation before routine use (92).

Gene editing and CRISPR-based molecular diagnostics, such as CRISPR-Cas13, have the potential to improve liquid biopsy sensitivity for low-abundance nucleic acid targets. CRISPR-Cas13 can enable highly specific RNA detection and may help identify resistance-related alterations promptly, supporting earlier therapeutic adjustments. CRISPR-based approaches can enhance the specificity and sensitivity of liquid biopsies by amplifying low-frequency biomarker signals, and they also support multiplexed assays that interrogate multiple targets in parallel (72, 73). These innovations are promising, but their clinical translation will depend on reproducible workflows, external quality assessment, and prospective evaluation of clinical utility.

Recent advancements in biomarkers and technologies such as exosomal biomarkers and gene editing are gaining recognition. Liquid biopsy may play a crucial role in personalized treatment; however, these methods require further clinical validation before widespread application. Liquid biopsies assist in combating drug resistance and improve patient care, and allow physicians to develop more effective cancer treatments.

### 6.3 Individualized precision treatment guidance

Liquid biopsy supports individualized precision treatment by enabling real-time tracking of tumor changes, therapeutic response, and emerging resistance. Minimally invasive sampling allows repeated profiling of ctDNA, CTCs, and EVs across treatment timepoints, capturing temporal and spatial heterogeneity that may be missed by single-site tissue biopsy (93). This feature is especially useful for identifying actionable alterations and resistance mechanisms that arise under therapy pressure, allowing clinicians to adjust regimens more rapidly. For example, in EGFR-mutant NSCLC, plasma detection of acquired T790M can guide subsequent targeted therapy selection (e.g., osimertinib), while recognizing that negative plasma results may require tissue confirmation in appropriate clinical contexts (4, 5, 54). Finally, combining longitudinal liquid biopsy data with computational models may improve prediction of therapeutic benefit and help reduce avoidable toxicities from ineffective treatments (37).

Besides tracking treatment efficacy, liquid biopsy is also being combined with other therapies, such as immunotherapy and targeted drugs, to enhance patient management. Identifying specific immune-related biomarkers through liquid biopsy can help determine which patients are most likely to benefit from immune checkpoint inhibitors, making treatment strategies more personalized for cancers like melanoma and colorectal cancer (94). Additionally, new treatments that use liquid biopsy, like adaptive therapy, allow for the continuous adjustment of treatment plans based on the current behavior of tumors, which enhances the precision of cancer care (95). As the field advances, combining liquid biopsy with other diagnostic methods, like imaging and histopathological evaluations, is anticipated to offer a more thorough approach to cancer treatment. This will ensure that therapies are customized not only to the tumor’s genetic profile but also to its evolving characteristics (51).

In conclusion, the application of liquid biopsy for directing personalized precision therapy represents a significant progression in the field of oncology. Liquid biopsy presents a significant opportunity to enhance patient outcomes through the dynamic monitoring of tumor progression and the timely adjustment of treatment strategies.

## 7 Conclusion

Collaboration among researchers and clinicians is essential, as it facilitates the integration of various techniques and insights from liquid biopsy studies. This collaboration will improve the validity of the findings. It will also aid in developing standardized protocols for global use in clinical settings.

Advancements in technology and multidisciplinary approaches are driving progress. Liquid biopsy is approaching standardization in clinical oncology. Future developments include multi-omics analysis, artificial intelligence, and innovative biomarkers. These advancements will improve the accuracy and efficacy of liquid biopsies in cancer treatment. These advancements can improve patient classification and refine treatment protocols. Outcomes may be better tailored to individual needs. Liquid biopsy has transformed cancer treatment and significantly improved disease monitoring. This progress aligns with technological advancements.

A comprehensive approach integrates genetic, epigenetic, and proteomic data. This integration enhances the development of a unified cancer care model. It emphasizes the importance of personalized treatment strategies. These strategies are tailored to the unique molecular features of each patient’s tumor.

In summary, liquid biopsy is nearing integration into cancer management, but it is essential to address current challenges and utilize various research perspectives that aid its advancement. Collaboration among researchers, clinicians, and technologists, along with the adoption of innovative methodologies, can enhance the application of liquid biopsy in precision medicine, resulting in more effective and personalized cancer treatments. The forthcoming journey is complex; however, with dedicated efforts and a strong commitment to progress in this field, we can anticipate a future where liquid biopsy significantly transforms cancer treatment.
